# Involving patients and the public in medical and health care research studies: An exploratory survey on participant recruiting and representativeness from the perspective of study authors

**DOI:** 10.1371/journal.pone.0204187

**Published:** 2019-01-07

**Authors:** Jonas Lander, Holger Langhof, Marie-Luise Dierks

**Affiliations:** 1 Institute for Epidemiology, Social Medicine and Health Systems Research, Hannover Medical School, Hannover, Germany; 2 Charité - University Medicine Berlin, QUEST - Center for Transforming Biomedical Research, Berlin Institute of Health, Berlin, Germany; 3 Institute for History, Ethics and Philosophy of Medicine, Hannover Medical School, Hannover, Germany; Cardiff University, UNITED KINGDOM

## Abstract

Research on patient and public involvement so far concentrates on defining involvement, describing its methods, and analyzing involvement practices in various individual research disciplines. There is little empirical data on the process of and aims for selecting (lay) PPI participants, and to what extend they can and should be representative of the population at large. To explore practices and perceptions on these issues and on future PPI conduct more generally, we sent an electronic survey to authors who published involvement activities as part of their studies in medical and social science journals. We identified such authors with a systematic search of five databases and applied descriptive statistics for analysis. Of those who returned the survey (n = 127 of 315; 40%), most had previously conducted involvement activities (73%). 45% reported more than one type of involvement, e.g. consultation and deliberation and participation (14%) and to have recruited more than one type of participant for their PPI activity (56%), e.g. ‘lay publics’ and ‘expert publics’ (33% of 71). Representativeness was often seen as a crucial objective when selecting PPI participants, while less than half found it very easy (9%) or rather easy (34%) to select participants. Many respondents considered achieving good representativeness difficult (52%) or very difficult (17%). They identified significant respective challenges and desired more guidance on various aspects of planning and conducting PPI (56%). 55% thought that the concept of “involvement” should be changed or improved. We conclude that recruiting lay people for PPI activities and deciding about and handling representativeness are controversial in current PPI practice, given the manifold challenges mentioned by the survey respondents. Our findings may inform further research particularly regarding–the potentially many cases of–unpublished PPI.

## Introduction

Recent research on Patient and Public Involvement (PPI) has identified a range of reasons to involve patients and the public in medical research and health policy. These include the need to align research priorities with societal preferences [1–3], the reorganization of existing health care services [4], and assessing the impact and value of health technologies and health services [5]. PPI may also increase transparency, legitimacy and accountability of scientists and policy-makers vis-à-vis society [6–8]. Others highlight its importance simply because so many decisions about health care are financed by tax payers, who should therefore have a stake in relevant decision-making processes [9].

Various (inter-)national organizations such as INVOLVE UK, NICE Patient and Public Involvement Programme (PIP) and the International Association for Public Participation (IAP2) run PPI advisory and interest groups. They serve different objectives and tasks, mainly advancing PPI frameworks, strengthening PPI in guideline development, and making PPI a prerequisite for instance for project funding [10–12]. Another common issue is the actual definition of PPI. While full consensus is yet missing, there is a tendency to distinguish several stages of active and passive involvement, i.e. a) informing/educating (least active), b) consulting for opinions and preferences, or c) involving lay people as ‘co-producers’ of research (most active) [1,13–15]. “Deliberation” is another commonly used term to refer to discussing and exchanging different perspectives on a given subject and, based on this, rethink one’s own position and adapt or explore new perspectives ultimately [16,17].

Besides such more general, conceptual issues, previous research has pointed to “practical concerns around defining and representing the community whose engagement is sought” [18] and the challenge of being clear about the actual “roles” [19] and “types” of patients and public [16]. An initial challenge concerns the terminology of ‘patients’ and ‘public’, and it is argued that there is a difference between these terms which is often not explicated [20,21] and a need to describe who exactly is involved [22]. For example, patients may want to influence the setting of research priorities to benefit their individual health. ‘The public’ may be more concerned about the amount of money invested in medical research generally. It is further argued that ‘public’ often lacks clarity, and that too many terms such as ‘citizens’, ‘service users’, ‘community members’, ‘lay people’, ‘carers’, and ‘tax-payers’ are used without explication [8,23]. While there could be clearer distinctions ideally, this may often be difficult in practice, and patients may be defined simply as a “subgroup of the wider role of citizen or member of the public” [21].

Related to these distinctions is the question about the representativeness of PPI participants. On the one hand, it may help to avoid the systematic exclusion of some social groups [8]. Also, recent PPI guidelines [22,24] and evaluation tools [1] describe the (careful) selection and description of participants as an important feature of ‘high quality PPI processes’. If participants of a specific PPI activity should be representative, various types can be differentiated, particularly quantitative/statistical (statistical reflection of socio-demographic characteristics, i.e. age, gender, etc.–referred to as “quantitative representativeness” hereafter), qualitative, (reflection of the broadest possible range of existing views), discursive (reflection of discourses on a given subject), and elective representativeness (electing certain representatives to act on behalf of others) [7,25–27].

On the other hand, depending on the precise aim and subject, PPI may require specific participants that cannot be regarded representative, for example patients with advanced disease-specific knowledge. Also, as long as PPI is a ‘side feature’ rather than an integral part of research, the research teams’ organizational and financial resources may not allow for highly sophisticated recruiting processes. Hence, achieving representativeness may often be difficult in practice [28–30] and may not always be desirable.

Various previous studies have assessed and debated PPI terminology and the challenges it entails [2,21,23], different types of representativeness and how they are used [29,31], and current PPI practices more broadly [18,32–34], using for instance debates, framework developments, and literature reviews. Few studies took a more practice-oriented approach by conducting interviews with professionals on PPI in general [35,36], and with small sample sizes [18,29]. The GRIPP2 checklist recommends to describe and transparently define the individual steps taken during PPI, such as the definition of PPI for that study and the methods used [22].

To our knowledge, this is the first study aiming to explore practices and preferences regarding selection of PPI participants, representativeness objectives, and PPI conduct from the perspective of authors of published involvement activities. Since previous research has outlined that determining if and how PPI participants can be representative and how to differentiate “representativeness” in different PPI contexts are challenging, it may be important to first provide an empirical overview of how the respective aspects are currently handled, in order to then begin with for instance consensus-finding initiatives on these and related controversial aspects. Lastly, the assessment of authors’ practices and preferences on the above-mentioned issues should also help understand in how far representativeness is important at all for meaningful involvement and, more generally, how satisfied authors are with the PPI concept.

## Materials and methods

### Data search

**Fig 1:**
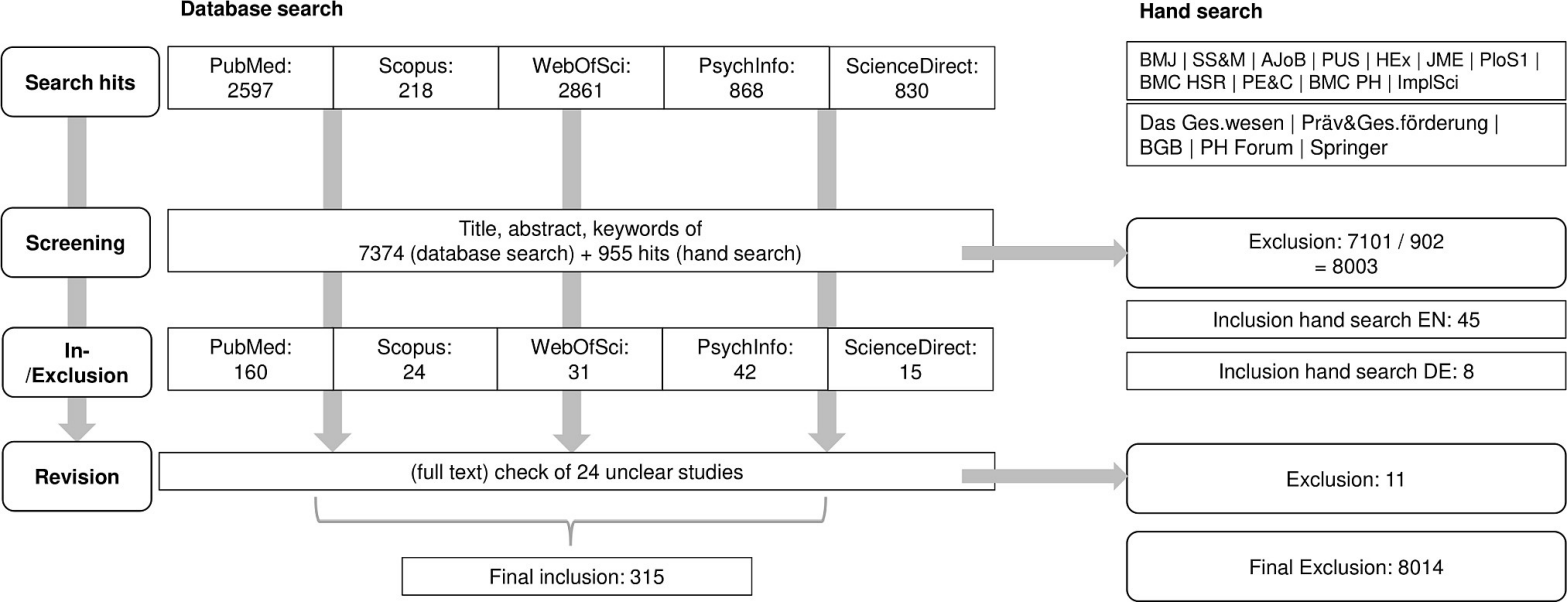
Database search.

This study has been approved by the Ethics Committee of Hannover Medical School (3465–2017). The data were gained via an online survey distributed to corresponding authors who published their PPI activities in the field of medical and health care research. While the perspective of public and patient contributors seems important, our survey was on specific (published) PPI activities, for which the authors are the primary (and often only locatable) contact point for questions on the planning and process. To gain the necessary contact information, we first conducted a systematic search for studies in relevant medical and sociological databases (Fig 1). For this, MeSH terms of a) PPI and b) medical research, healthcare research and health policy were combined into search queries, which we adapted to the respective database where necessary (Table 1).

**Table 1:** Search terms.

Database	Search term
PubMed	((((((((((("Medical Research") OR "Translational Medical Research"[Mesh]) OR "Clinical Medicine"[Mesh]) OR "Regenerative Medicine"[Mesh]) OR "Precision Medicine"[Mesh]) OR "Biotechnology"[Mesh]) OR "Biomedical Research"[Mesh]) OR "Health Policy"[Mesh] OR "Health Policy Development" AND "Consumer Participation"[Mesh]) OR "Public Engagement") OR "Public Involvement") OR "Public Deliberation") OR "Public Consultation"
PsychInfo	exp Involvement/or exp Community Involvement/ or “Public Involvement”.mp. or “Public Participation”.mp. or exp Public Opinion/ or “Public Consultation”.mp. and exp Medical Sciences/or “Medical Research”.mp. or exp Health Care Policy/ or “Health Policy”.mp. or “Health Policy Development”.mp.
Scopus	(TITLE-ABS-KEY ("Public Participation") OR TITLE-ABS-KEY ("Public Involvement") OR TITLE-ABS-KEY ("Public Consultation") AND TITLE-ABS-KEY ("Medical Research") OR TITLE-ABS-KEY ("Healthcare Research") OR TITLE-ABS-KEY ("Health Science") OR TITLE-ABS-KEY ("Health Policy")) AND DOCTYPE (ar OR re) AND SUBJAREA (mult OR agri OR bioc OR immu OR neur OR phar OR mult OR medi OR nurs OR vete OR dent OR heal OR mult OR arts OR busi OR deci OR econ OR psyc OR soci) AND PUBYEAR > 2009
ScienceDirect	pub-date > 2009 and "public participation" OR "public involvement" OR "public consultation" OR "public engagement" OR "public deliberation" OR "consumer participation" AND "medical research" OR "medical science" OR "health policy" OR "translational medical research".
WebOfScience	TOPIC: ("Public Involvement") OR TOPIC: ("Public Participation") OR TOPIC: ("Public Consultation") OR TOPIC:("Consumer Participation") ANDTOPIC: ("Medical Research") ORTOPIC: ("Healthcare Research") ORTOPIC: ("Clinical Medicine") Refined by: RESEARCH DOMAINS: (SCIENCE TECHNOLOGY OR SOCIAL SCIENCES) AND RESEARCH AREAS: (HEALTH CARE SCIENCES SERVICES OR BIOMEDICAL SOCIAL SCIENCES OR SOCIAL ISSUES OR SOCIOLOGY OR SOCIAL SCIENCES OTHER TOPICS OR MEDICAL ETHICS OR RESEARCH EXPERIMENTAL MEDICINE OR SCIENCE TECHNOLOGY OTHER TOPICS OR REPRODUCTIVE BIOLOGY OR LIFE SCIENCES BIOMEDICINE OTHER TOPICS OR INTEGRATIVE COMPLEMENTARY MEDICINE) AND DOCUMENT TYPES: (ARTICLE OR REVIEW) Timespan: 2009–2016. Search language = Auto

We included publications if they a) directly reported on PPI as part of a research project, b) in medical or health care research, c) between 2009 and 2016, and d) in English or German. Regarding a), we framed PPI as spectrum of activities with varying degrees of active and passive involvement (see introduction). This was done since a) different researchers and other PPI actors have different understandings of what involvement is and hence also use different terminology, e.g. involvement, engagement, participation, deliberation, and b) the reporting on PPI is neither mandatory nor standardized yet, making it difficult to focus on one type alone.

A sample of 800 search hits was screened separately by each of two researchers (HL, JL) to achieve coherence regarding the in- and exclusion of studies. The screening results were compared and minor differences were resolved by discussion. We also discussed studies that did not show clearly how the input gathered could be used to develop or adapt research or decision-making processes and excluded some. For instance, one study reported experiences of women with cancer diagnosis, but was unclear about the translation of this input into practice. We also hand-searched those journals that yielded at least 3 published PPI activities in the database search (n = 11).

### Data-collection

From the included studies, we collected the necessary information about the corresponding authors, including name, position, and email address. This information was often updated as authors had moved institution or changed their contact details. The Ethics Committee waived the need for separate consent as no personal data were collected and all information was provided to survey recipients in advance.

The survey included questions with single and multiple answers on a) authors’ previous experiences with PPI, b) specific questions on underlying aims regarding participant selection, characteristics and representativeness, and c) more general preferences for future PPI (S1 Table). For details on the survey design, piloting, approval and administration, see the adapted CHERRIES checklist for the reporting of electronic surveys from [37] (S2 Table).

Authors were pre-notified of the research project via email one week before the actual survey. We asked the corresponding authors to forward the survey to co-authors or send us their contact details if they could answer the questions better. During an eight-week period of collecting responses (March–April 2017), two follow-up reminders were sent to elicit further answers.

### Data analysis

The data were collected via SurveyMonkey, and exported to SPSS Statistics for analysis of frequencies, correlations between answers to different questions–for instance recruiting aim and recruiting methods–and assessment of multiple answers (using multiple answer sets and cross tables). Since there may be differences in how ‘actively’ participants a are involved in different types of PPI, we compared the frequencies of answers separately for studies that aimed either only at “consultation” (less active) or only at “participation”/ “deliberation” (more active) for those aspects where differences may be most obvious: recruited participants (Q. 3, 7), importance of representativeness (Q. 4), representativeness objective (Q. 5), recruiting methods (Q. 6), PPI methods (Q. 9), achieving representativeness (Q. 13, 14), and guidance/changes on “PPI” (Q. 15, 17).

Regarding the analysis of free-text answers (Q. 19), two researchers (JL, HL) independently categorized answers deductively according to the main survey items (selection of participants, representativeness objectives and outcomes, challenges associated with participant selection and representativeness, and role and relevance of PPI). The categorization was then compared, and differences and unclear items discussed with a third researcher (MLD) to reach agreement.

An a priori quality assessment of selected studies was not applicable since the focus was on authors’ practices, not on the reporting.

## Results

### General characteristics of studies

Of 8,329 search hits, 315 were eventually selected based on the criteria stated in 2a). The final response rate was 40% (n = 127). Since the survey was anonymous unless authors voluntarily provided the publication title, the PPI subject could only be obtained for 65 studies (Table 2).

**Table 2:** General information about included studies.

Subject (n) ª	Genetics, genomics (10)	Biobanking, biobank research (6)	Priority setting (3)	Cancer research / treatment (3)	Research, clinical trials (4)	Decision-making and change (3)	End-of-life care (2)	Quality, quality indicators (2)	Research participation (2)	Other ᵇ (29)
**PPI aims (n)**	Consult-ation only (40)	Partici-pation only (7)	Deliberation only (8)	Consul-tation + deliberation (8)	Consultation + participation (10)	Other combination with 2 aims (7)	Consult. + delib. + particip. (8)	Info. + consult. + delib. (8)	Info. + consult + delib + particip. (8)	Other combinationwith ≥ 3 aims (14)
**PPI methods (n)**	Question-naire only (16)	Interview only (6)	Focus group only (8)	Assessment only (0)	Citizen jury only (1)	Question—naire + interview (10)	Question-naire + focus group (9)	Other combinationwith 2 methods (21)	Information + question. + interview + focus group (4)	Other combinationwith ≥ 3 (47)
**Type of partici-pants (n)**	Lay publics only (26)	Expert publics only (4)	Lay patients / lay publics only (13/6)	Family members or represent-atives or medical staff only (1)	Others only (6)	Lay + expert publics (4)	Lay + expert patients (6)	Other combinationwith 2 types (9)	Lay publ. + expert publ. + lay pat. + expert pat. + family members + repr. + med. Staff (5)	Other combination with ≥ 3 (40)
**No. of partici-pants (n)**	Up to 10 (10)	11–20 (25)	21–50 (15)	51–100 (14)	101–200 (10)	201–500 (18)	> 500 (30)			
**Dura-tion PPI (n)**	Up to 1 day (78)	1–2 days (13)	2–5 days (30)	> 5 days (0)						

**ª** only those studies for which the authors voluntarily mentioned the study title (n = 65)

ᵇ Autism research, Long-term conditions, Drug licensing, Pharmacy alcohol screening, Depression, Consent for emergency research, Rheumatology, Surgical research, Grief, Ageing care, Dietetic consultations, Drug advertisement, Vaccine safety, Intermediate pulmonary nodule, Cerbral palsy, Diabetes, Organ donation, Nanotech, Dementia, Sensitive health topics, Health tech design, Mental health, Dengue control, Embryo status, Degenerative ataxis treatment, Biomedical research, General practice, Physician-pharmacy interaction, Health insurance.

The majority of authors had conducted PPI before (73%); most of them (46%) had been involved in between two and five PPI activities. 21 authors said they had already conducted six to ten or even more activities.

Across all 127 PPI activities, consulting for preferences, opinions, etc. was reported most often as the sole aim (31%). Deliberation and participation were reported seven and eight times respectively as the sole aim. All others combined different aims, e.g. consultation *and* participation (8%). While deliberation and participation were rarely reported alone, they were often reported alongside other aims (deliberation 39%; participation 33%).

The most often reported single methods used to achieve these aims were questionnaires (13%), interviews (5%), and focus groups (6%). The most frequent combinations were questionnaires and interviews (8%), questionnaires and focus groups (7%), and information *and* questionnaires *and* interviews *and* focus groups (6%).

**Fig 2:**
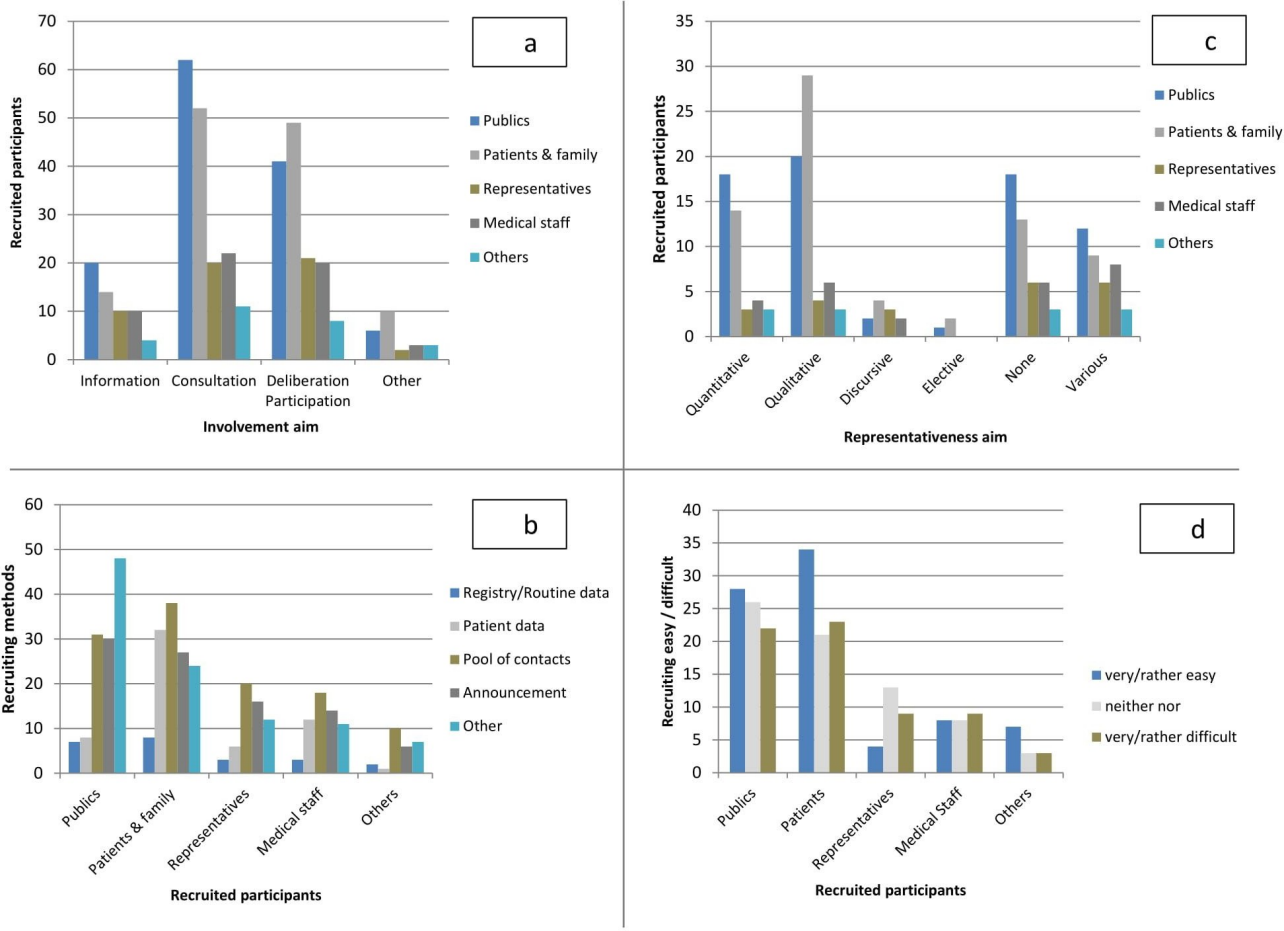
Summary of results.

Although authors mentioned different involvement aims, there was no particular difference in how often different methods were used, i.e. most often questionnaires/interviews, then focus groups, information, and assessments/juries, with “other” methods in last place (Fig 2A).

### Aims for recruiting participants and handling representativeness

50 PPI activities recruited only one type of participant, most often lay publics (20%) or lay patients (10%). Studies that recruited only one of expert publics and patients–i.e. patients/publics who identify as having advanced knowledge of the subject for which their involvement is sought, in contrast to ‘lay’ participants who do not consider themselves to have any subject-specific knowledge–or family members (of patients) and representatives (of an organized group, e.g. patient advocacy groups) were much rarer. 71 reported recruiting a combination of two or more. These included lay and expert publics or patients, and lay and expert patients with family members and representatives. 5 reported a combination of 6 different types of participants, such as lay and expert publics, lay and expert patients, family members, and representatives. All other combinations were reported once each (Table 2). Regarding the comparison of consultation and participation/deliberation approaches, the latter recruited lay and expert patients and family members more often (46% respectively) than the former (33%, 23, 19%), while lay publics were recruited more frequently for consultation (67%) as for participation/deliberation (38%).

Recruitment methods were similar for the various participants: most often from a pool of contacts, then announcements, patient data, “other” methods, and least often registry/routine data (Fig 2B). Only for lay and expert publics were “other” methods used almost twice as often as when recruiting patients, representatives, etc. Most frequently (3 or more mentions), “other” methods were described as direct approaching at community events, public calls for interest in community groups, social media and subject-specific websites, random contacting via an external agent, and organizations with frequent contact to community members or patient/public representatives. Consultation and participation/deliberation activities differed somewhat in the recruiting via patient data (38% vs. 23%), contact pools (41% vs. 21%), and other methods (41% vs. 54%).

Many authors rated representativeness of the participants vis-à-vis the general population very (33%) or rather important (38%). Those that aimed solely at consultation or deliberation/participation accorded representativeness of equal importance (rather/very important: 75%, 75%). 17% of all authors, however, indicated that representativeness was rather not or not at all an important characteristic. When selecting PPI participants, most authors either aimed for quantitative (25%) or qualitative representativeness (32%). Within the subgroup of deliberation/participation, slightly more aimed at qualitative (35%) and quantitative representativeness (30%) compared to the overall sample (32%; 25%). Also, deliberation/participation activities less often had no representativeness aim (13%; overall sample 20%). Discursive and elective representativeness were reported only rarely (4%; 2%) and 19% of all respondents did not indicate any aim.

Among the various representativeness aims, the distribution of (types of) participants was similar: most often lay/expert publics, then lay/expert patients, medical staff, representatives, and last “others” (Fig 2C). However, lay/expert publics were reported most often for quantitative, and lay/expert patients for qualitative representativeness.

### Outcomes of the recruitment process and levels of representativeness achieved

55 respondents mentioned that recruiting the intended group was very easy (9%) or rather easy (34%). 36 found it to be neither easy nor difficult (29%), rather (28%) or very difficult (2%). The recruiting was more often rated very/rather easy for consultation than for participation/deliberation (58% vs. 33%; overall sample: 44%).

Recruitment was never mostly found to be easy: there were almost as many “rather/very difficult” ratings as “rather/very easy” ratings for each type of participant (Fig 2D). Only for recruiting lay/expert patients and their family members, who were most often recruited via a contact pool and patient data, did recruitment seem somewhat easier. Apart from the difficulties during the selection process, most indicated success in finding the intended participants eventually (86%).

Besides the conclusions respondents drew from their respective involvement activities, we also asked for their more general perspective on representativeness. Here, 71% said that representativeness is generally rather (40%) or very important (31%). Only 5% found it rather or very unimportant; all others were undecided. Representativeness was seen almost equally important among consultation and participation/deliberation (very/rather important 82% vs. 76%).

In contrast, actually achieving representativeness was perceived to be harder– 69% viewed this as rather (52%) or very difficult (17%). Even among those who saw representativeness as (very) important, a considerable part indicated that it is generally rather not (n = 40) or not possible to achieve (n = 8). Achieving representativeness was perceived slightly more difficult for consultation compared to deliberation (73% vs. 66%).

Regarding the relevance of (future) guidance for conducting PPI, 31% think that more guidance is needed on overall planning and conduct. Guidance for selecting participants was desired by 28% of respondents, who often looked to research organizations for this. As for the difference between consultation and deliberation/participation, authors of the latter desired overall guidance more often (36%) than authors who aimed at consultation (21%).

Further, more than half of all authors (55%) indicated that the concept of PPI as such requires modification. Those aiming at deliberation/participation agreed more often (59%) than the overall sample (55%).

Finally, 37% felt that the terms “patients” and “public” need to be better defined or differentiated. Within the group of deliberation/participation, authors were happier with the terminology: only 23% would welcome changes.

### Open answers on the relevance and challenges of recruiting participants and achieving representativeness

45 of 127 respondents answered the open-ended question “Based on your experiences, is there something regarding the selection of participants or the role and relevance of representativeness of participants that you want to add here?” The analysis allowed for a topic-wise categorization of answers (S3 Table).

A large part of the comments was made regarding challenges and issues to consider when selecting PPI participants. For instance, values, orientations, and topic-specific opinions may be as important as socio-demographic characteristics. More generally, much more effort and “hard thinking” is needed to select participants appropriately than to simply replicate the wider socio-demographic range. It was also stated that selecting participants should be aligned more thoroughly with the PPI aims and, related to this, that “the difference between patients and publics be understood” (Box 1).

**Box 1. Extracts from free-text answers**

**Representativeness**

“The importance of representativeness of participants strongly depends on your project/research objectives.”

“It is essential to high quality research. There is much guidance on PPI already available, however, there is no harm in receiving more to ensure we do it better.”

“The notion of representativeness in PPI is a load of nonsense and entirely misses the point. Talking about representation deflects from the real value of PPI. No one questions how representative I the researcher am when I conduct a project. Why do we need to question how representative patients are? It's time to move away from this and get on with involving patients in our work.”

**PPI participants**

“I think it is important that any patient and public involvement be clearly identified in regard to the research questions and aims. Specifically, why do you want to speak with or engage with this group and how will their contributions help with the research.”

“It is essential that the differences between patients and publics be understood. As well, we need a third category—"community" which identifies a different public constituency with collective interests that also warrant engagement and representation.”

**Limitations and challenges**

“It's not a good idea to use the same people for PPI to represent a group on different issues. The PPI group should have expertise in the area or topic, and the same people could not be in PPI.”

“'Representativeness' should be approached with extreme caution as it is rare for people to agree on what participants should be representing: demographics, perspectives, the 'everyman', the experiential expert etc., and thus it can be manipulated.”

Regarding representativeness, several comments were made about how it is understood and what representativeness means, e.g. “everyone [has] a different view of what representative participants would be”. Authors also pointed to how representativeness differs in different types of research, and that a more general decision needs to be made about whether PPI aims to represent (i.e. ‘include’) views and perspective of various societal groups–without these views necessarily being representative of the population at large–or whether a correct statistical reflection of all population characteristics is required (e.g. comments a) 4, a) 7, c) 1, c) 6, d) 5, d) 11 in S2 Table). It was further mentioned that the concept entails more “nuances” beyond broader categories such as qualitative and quantitative representativeness.

As for the relevance of representativeness, some authors opined that it is indeed an important prerequisite for successful PPI (see first part in Box 1). Also, “the need for representativeness varies based on [different] studies”. However, many indicated that representativeness is rather not important/relevant or challenging in practice, not least given the difficulties of finding a common definition and implementing it. Three respondents argued that focusing on representativeness may distract from other, more important issues in PPI, and that the notion may easily be misleading, e.g. it “deflects from the real value of PPI”. Another respondent suggested that representativeness may simply not be desirable, for instance when being interested in “a range of perspectives” and “views” instead.

## Discussion

### Relation of main findings to previous research

Besides those previous studies that discussed recruitment and representativeness from a theoretical perspective or addressed them very generally in reviews, few have dealt with this subject in detail and/or used a similar methodology. [35] and [36] investigated industry and researchers’ attitudes towards PPI using interviews (n = 15; n = 21), though without any particular focus on recruitment and representativeness. A recent study by South et al. [38] describes the kind of “representatives” involved in a small sample of UK-based case studies, though without further (quantitative) analysis regarding recruiting or representativeness.

Others assessed how representativeness is understood in different contexts using qualitative interviews [29], and current challenges from the perspective of research network members [18]. While the latter did not address representativeness per se, they even speak of a “crisis of representation”. Further, the RAPPORT study [39] assessed current PPI from the perspective of UK-based principal investigators, though without any specific focus on the handling of recruiting and representativeness. A Delphi process by Kearney et al. [40], which involved PPI stakeholders from a variety of backgrounds, describes recruiting and retention of participants as a PPI research priority, however in relation to PPI as a means to improve the recruiting for clinical trials, not PPI participants as such.

While these studies frame recruitment and representativeness as central issues, we aimed to explore how these aspects are handled in practice by one of the principal groups concerned with PPI, i.e. study authors. It may be easier to deal with these issues once actual practices are known.

While many authors had had prior experience with involving lay people in research, they often would still welcome more guidance; hence, previous experience may not automatically help researchers deal with trickier matters such as determining whether and what type of representativeness is necessary. Researchers may also find it difficult selecting ‘representative’ participants if their research community does not per se have (easy) access to those who are more difficult to recruit, i.e. vulnerable populations.

A considerable number of studies recruited more than one target group (38% at least 3), often mixing up distinct types of participants. While including participants with different backgrounds may increase spectrum of perspectives, one of the current challenges is to better define and differentiate whom to involve in research, and how, which has also been mentioned in the free-text answers. Also, few of those studies for which authors gave the title provided an explanation for why specific participants were included or what “public” etc. means in their case, other than to provide general sample information. Further, while respondents often seemed overall positive about their own study, many (Q. 19) expressed conceptual and practical difficulties with differentiating PPI participants, and what representativeness means in different contexts.

Regarding representativeness, some previous studies mention that defining its meaning and agreeing on the nature of representativeness more generally (e.g. [32,41] still requires clarification. However, particularly the free-text answers in our survey show that its relevance is questionable. While some see it as a crucial aspect, others doubted its necessity, not least given the pertaining challenges of defining and achieving representativeness. Because of the various critical perspectives, it could also be argued that representativeness is not something that should receive particular attention in PPI, but rather in those types of research where sampling, i.e. in- and exclusion criteria are definitely required (and hence conclusions about representativeness can be drawn more easily).

Besides considering the relevance of representativeness, the survey shows that its meaning often differs according to the respective PPI aim and selection of participants. Most obviously, the free text answers suggest that representativeness is not always meant to ensure correct reflection of socio-demographic population characteristics, but the (qualitative) inclusion of experiences and perspectives from different societal groups and individuals more generally, which may be referred to as ‘representation’ rather than ‘representativeness’. One respondent for instance commented that “selection of participants should not be driven solely by […] randomness but should use aspects of qualitative judgement also”. A potentially helpful way to think of the meaning of representativeness was expressed by one respondent stating that “both a geographical community and a disease-specific community” should be considered when selecting PPI participants, or, depending on the specific aim, only one of those. However, since several authors pointed at the need to adapt participant selection according to the PPI aim, the need to provide reasons for why certain ‘patients’ or ‘public’ qualify for PPI remains, regardless of their representativeness function.

Since PPI activities are not solely determined by participant representativeness, which often seems to create difficulties for finding participants as outlined in various survey responses, social science research argues that other concepts such as inclusiveness may be employed instead. Aspects such as individuals abilities and resources to influence decision-making, the organizational context that enables or hinders individuals’ participation and impact, the professionalization of participants, and the actors’ networks and changing power relations are influential on PPI, too [42–44]. This assumption is supported in different responses to our survey, stressing the boundaries of representativeness.

### Methodological considerations

Firstly, the survey mainly relied on close-ended (and thereby shorter) questions to assess PPI practices from a broader group of authors as a basis for further, possibly more detailed analyses. Particularly the responses to the free-text question showed that some issues could be addressed in a more qualitative, dialogue-based way beyond close-ended questions.

To reach as many authors as possible, we conducted a database search and did not limit this by discipline, allowing for insights into PPI practices across a range of research areas. However, we could not conduct a subgroup analysis regarding potential differences for example on recruiting objectives across different research topics as the survey was anonymous and hence, retracing all topics was not possible.

Further, as the reporting on PPI and particularly the active types of involvement is neither mandatory nor standardized [14], we applied a rather broad definition. More research is needed to identify potential differences regarding PPI participants across the different types of involvement.

While it may generally be important to differentiate types of PPI, our objective was to assess participant selection and representativeness practices. This may be equally relevant both for more and less active involvement, as decisions about the how and why of selecting participants apply in either case. Nevertheless, PPI may take place in various academic and non-academic settings, and its methodology may hence differ, as well as the individuals and groups responsible for the planning and conduct. Since we contacted PPI organizers based on publications in medical and social science journals, we could not include involvement efforts not published along research projects. It may be vital to enhance our findings by analyzing a) PPI activities published or reported in different formats and b) cases where PPI was not published.

### Conclusion

The results of our survey highlight several challenges, namely deciding whether or not representativeness is actually required according to the involvement aims, and if so, which ‘type’ may be most suitable, defining and differentiating among different ‘types’ of participants, justifying why some were chosen and others not and, more generally, distinguishing ‘patients’ and ‘public’. Also, while (some) PPI guidance is available from research and institutions, several survey respondents mentioned the need for more guidance and a better understanding about “involvement”. Hence, available support may not have been a specific help for many of our respondents. However, we could not follow-up if authors who already used PPI guidance would still welcome more/other guidance, which may be important for adjusting already available materials to specific needs.

In view of the practical difficulties of selecting participants as well as the lack of reporting on PPI activities (see below), those planning and conducting PPI may consider the following aspects: a) providing reasons for why certain participants were selected and how the selection fit the recruiting aims, b) making the type and degree of representativeness of participants dependent on the involvement aim and not seeing representativeness as a prerequisite, c) defining the role of participants in the involvement process, how they are involved, and thinking about potential implications for selecting participants, d) considering whether the experiences, skills and qualifications of the research team suffice to plan and conduct PPI, and e) being clear about how potential recruiting and representativeness limitations impact on the process and results.

Since our findings and the named challenges only count for the views and practices of study authors who published PPI activities as part of their research, it seems relevant to assess how the named challenges are handled when PPI is not published. This may also reveal insights about whether reporting on PPI and particular aspects such as participant recruiting is lacking so far [31] because authors simply do not consider it relevant, or because of actual difficulties with PPI conduct in practice.

Lastly, as various theoretical and practice-oriented contributions to PPI research are now available, future research may also focus on consensus-finding approaches to the pertinent challenges, which, in view of the complexities around recruiting and representativeness mentioned by survey respondents, should go beyond thinking of researchers/research groups as the only ones to determine and bear the responsibility for the planning and process of PPI. This argument is support by recent guidance on ‘co-producing’ research projects, in which the sharing of roles and responsibilities in the planning and conduct of involvement, i.e. co-production of research by researchers and publics/public representatives is a key aspect [45]. The involvement of lay people in further research about PPI seems highly relevant, not least since our survey focused (only) on the perspective of researchers. In particular—while the survey aimed to provide a basis of evidence regarding selection of participants and handling of representativeness in PPI–further work may be necessary to unfold aspects such as reasons for why and how certain PPI participants/contributors qualify to represent others, how to be clear if representativeness is necessary or not and, based on the latter, alternatives to representativeness and respective guidance on PPI methodology.

## Supporting information

S1 Table Survey. (PDF)

S2 Table CHERRIES checklist. (DOCX)

S3 Table Categorization of open answers to question 19. (DOCX)
