# Supplementary material for: Involving patients and the public in medical and health care research studies: An exploratory survey on participant recruiting and representativeness from the perspective of study authors
Source: PLoS One. 2019 Jan 7;14(1):e0204187. doi: 10.1371/journal.pone.0204187 (PMC6322864; doi:10.1371/journal.pone.0204187)
Supplement: S1 Table — (PDF) [file pone.0204187.s001.pdf]

Survey:
Patient and Public Involvement in medical research and health policy: Who are the participants and what is their role in the study?

(Introductory text)

Part 1

1. Have you conducted or been involved in PPI before the study we refer to in the e-mail?
a) No
b) Yes, in about ______ studies

2. Whom did you intend to recruit for your PPI? (tick all that apply)
a) 'Lay publics' without topic-wise previous experiences
b) 'Expert publics' with topic-wise previous experiences
c) Lay-patients without topic-wise previous experiences
d) Expert-patients with topic-wise previous experiences
e) Family members (of patients)
f) Representatives of an organized group, e.g. a patient organization or self-help group
g) Medical staff/professionals
h) Others, namely: ____

3. Was it important for your study that the participants would be representative – regardless of whether representativeness was reached eventually?
a) Yes, very important
b) Yes, rather important
c) No, rather not important
d) No, not important

4. If yes, have you set a specific aim regarding the representativeness of participants beforehand?
a) Quantitative/statistic representativeness (statistical reflection of sociodemographic characteristics)
b) Qualitative representativeness (reflection of a spectrum of views)
c) Discursive representativeness (reflection of different discourses on a subject)
d) Elective representativeness (representatives elected to represent for others)
e) No, representativeness was not a central criterion

5. How did you recruit participants? (tick all that apply)
a) Via registry data (e.g. registration office)
b) Via routine data/records (e.g. health insurance company, accident insurance)
c) Via patient data from a medical institution (e.g. hospital)
d) Via a pool of contact from an association/organization (e.g. patient organization, self-help group)
e) Via a public announcement/advertisement (e.g. newspaper, Internet)
e) Differently: ____

6. How difficult/easy was it to recruit 'suitable' participants?

a) Very easy
b) Rather easy
c) Neither easy nor difficult
d) Rather difficult
e) Very difficult

7. Did you manage to recruit the intended group of participants eventually?

a) Yes
b) No
c) This wasn't checked exactly during the study

Part 2

The following four questions relate to the actual conduct of your PPI activity.

8. How many participants took part in your PPI activity?

a) less than 10
b) 10-30
c) 31-50
d) 51-100
e) 101-200
f) 201-500
g) more than 500

9. How much time did it take for the patients/public to participate?

a) 1 day or less
b) 1-2 days
c) 2-5 days
d) more than 5 days

10. Which method(s) have you used for your PPI activity? (tick all that apply)

a) Information (e.g. presentation, written material)
b) Questionnaires, surveys (written, digital)
c) Interviews (in person, by telephone)
d) Focus-groups, discussion rounds
e) Assessments, classifications, rankings (e.g. discrete choice experiment)
f) Citizen jury, feedback-/advisory groups
g) A different method: ____

11. What was your aim when using these methods? (tick all that apply)

a) Patient/public information: informing, educating patients and public
b) Patient/public consultation: assessing, consulting for opinions, preferences, etc.
c) Patient/public deliberation: enabling patients/public to discuss and evaluate different perspectives on a given subject to come to an own perspective

d) Patient/public participation: actively involving patients/public in developing or adapting research- or decision-making processes
e) A different aim: ____

Part 3

In addition to the above questions regarding the aforementioned study, we would now like to learn something about your general attitudes and future preferences regarding the selection of participants for PPI and PPI in more general.

12. From your perspective, how important is it for PPI participants to be representative vis-à-vis the general/reference population?
a) Very important
b) Rather important
c) Rather not important
d) Not at all important

13. Do you think representativeness of participants is possible in every PPI activity?
a) Yes, always
b) Yes, usually
c) No, rather not
d) No, not at all

14. For future PPI activities, would you like to have more guidance? (tick all that apply)
a) Yes, for recruiting participants
b) Yes, for the overall conduct of PPI
c) Yes, for dealing with the results
d) No, this is not necessary
e) I don't know

15. Who should provide such guidance? (tick all that apply)
a) Research organizations
b) Policy organizations (governmental bodies or NGOs)
c) Different organizations: ____
d) I don't know

16. Should the concept of "Patient and Public Involvement" be defined more exactly in the future? (tick all that apply)
a) Yes, there should be a better definition of what is meant by "involvement"
b) Yes, "patients" and "public" should be differentiated more explicitly
c) No, "Patient and Public Involvement" is a sound concept
d) I don't know

17. Do you intend to do more PPI activities in the future?
a) Yes, certainly
b) Yes, maybe
c) No, rather not
d) No, certainly not
e) I don't know

18. Based on your experiences, is there something regarding the selection of participants or the role and relevance of representativeness of PPI participants that you want to add here?
Open answer

19. At last, you would us with the allocation of answered surveys if you name the title of the publication we referred to in the e-mail. The information is of course voluntary and all data will be treated confidentially.

____
