# Supplementary material for: Involving patients and the public in medical and health care research studies: An exploratory survey on participant recruiting and representativeness from the perspective of study authors
Source: PLoS One. 2019 Jan 7;14(1):e0204187. doi: 10.1371/journal.pone.0204187 (PMC6322864; doi:10.1371/journal.pone.0204187)
Supplement: S2 Table — (DOCX) [file pone.0204187.s002.docx]

**Supplementary file 2.** CHERRIES Checklist

| **CHERRIES Item Category** | **CHERRIES Checklist Category** | **Handling / Compliance** |
| --- | --- | --- |
| 1. Design | Describe survey design | Target population: researchers (authors) who have published PPI activities in peer-reviewed journals; Researchers/authors were selected in advance with a systematic search for respective publications in five databases (see Method section)  Type of survey: close and open-ended questions |
|  | IRB approval | Provided by Hannover Medical School (Reference number 3465-2017) |
|  | Informed consent | All relevant information was provided to survey recipients in advance: topic, objectives, length of survey, storage of data, voluntariness; anonymity; investigators; contact person |
|  | Data protection | No personal information was collected from survey respondents |
| 2. Development and pre-testing | Development | Development of questions: Questions were developed based on previous research findings and challenges stated as part of these research endeavours, particularly discussions on whom to involve in PPI (O’Shea et al. 2016), missing descriptions and differentiations among different types of participants (Frederiksson and Tritter 2017), relevance and feasibility of achieving representativeness (Snape et al. 2013, Martin 2012, Longstaff & Burgess 2010, Martin 2008), and recent understandings and varying definitions as well as available guidelines for researchers to plan and conduct “patient and public involvement” (Abelson et al. 2015, Staniszweska et al. 2017). Further, we applied questions and topics from a previous analysis by our research group on the reporting of PPI aims and methods (Lander et al. 2014) and recruiting and representativeness aims and outcomes in published PPI activities in the field of biomedical research (Lander et al. 2016). |
|  | Pre-testing | We piloted an initial version with a group of six researchers experienced in survey methodology and/or the survey subject who otherwise had no formal role in study to ensure neutrality. These researchers have also been involved in planning and organizing (own) PPI activities previously to ensure similarity with the target group. We used the online tool to send the survey to each participant and deleted responses before actual start of the survey. Each participant answered the questions and provided face-to-face feedback (comprehension probing) afterwards on a) technical functioning, b) wording, and c) overall structure, content and focus. Responses were collected and discussed with the research team. The main changes included rewording the introduction, deleting less relevant questions, rewording individual terms such as “PPI study” to “PPI activity”, re-ordering of questions and introducing page breaks, providing more detail for some of the questions (Q. 2, 4, 11, 12), specifically examples for the different types of PPI (Q. 12), differentiating “representativeness” in subcategories (Q. 4), differentiating target groups (Q. 2) and reframing the free-text question. A revised version was tested again to understand changes and to estimate the time needed to complete all questions. Lastly, all participants agreed to the final version. |
| 3. Recruitment process and description of the sample having access to the questionnaire | Open survey versus closed survey | Closed survey |
|  | Contact mode | Via E-Mail |
|  | Advertising the survey | None (selection of survey recipients via search for publications in scientific database, see 1.) |
| 4. Survey administration | Web/E-Mail | Link to survey provided in E-Mail; Data were stored automatically via the survey software and then exported to MS Excel / SPSS |
|  | Context | Professional online survey tool (SurveyMonkey) |
|  | Mandatory / voluntary | Voluntary |
|  | Incentives | None, except for mentioning respondents’ valuable contribution to research by answering the survey and receiving results after the study has been finalised |
|  | Time / date | 8-week period (March – April 2017) |
|  | Randomization of items or questionnaire | Not relevant |
|  | Adaptive questioning | Not relevant |
|  | Number of items | 20 |
|  | Number of screens | 3 excluding start screen |
|  | Completeness check | Respondents could skip questions when not knowing the answer, the options “I don’t know” or “something different” were provided were relevant. |
|  | Review step | Provided as part of the survey tool |
| 5. Response rates | Unique site visitor | No, only available with a costly update. This was also not relevant as the survey was only sent to a pre-defined group. |
|  | View rate (Ratio of unique survey  visitors/unique site visitors | As above |
|  | Participation rate (Ratio of  unique visitors who agreed to  participate/unique first survey  page visitors) | As above |
|  | Completion rate (Ratio of users  who finished the survey/users  who agreed to participate) | 127 completed surveys from 315 total surveys sent to study authors (response rate = 40%) |
| 6. Preventing multiple entries from  the same individual | Cookies used | Automatic feature provided by the survey tool; surveys could only be answered once from the same IP |
|  | IP Check | See above |
|  | Log file analysis | - |
|  | Registration | Once the survey was completed by a single user, the same IP address could not be used anymore to fill in the survey again. Researchers also have an own interest in preventing potential response bias. |
| 7. Analysis | Handling of incomplete questionnaires | One questionnaire was exempted from analysis; for those who skipped only few individual questions the respective item was understood as missing in the analysis. |
|  | Questionnaires submitted with  an atypical timestamp | - |
|  | Statistical correction | Both completed and incomplete surveys (single questions) are analysed; uncompleted questions are understood as missing. |
