# Supplementary material for: Involving patients and the public in medical and health care research studies: An exploratory survey on participant recruiting and representativeness from the perspective of study authors
Source: PLoS One. 2019 Jan 7;14(1):e0204187. doi: 10.1371/journal.pone.0204187 (PMC6322864; doi:10.1371/journal.pone.0204187)
Supplement: S3 Table — (DOCX) [file pone.0204187.s003.docx]

**Supplementary file 3**

Categorization of open answers (question 19)

|  | Category | Quote | N |
| --- | --- | --- | --- |
| a) | Conception of representativeness | 1. Representativeness means such different things in qualitative and quantitative designs. 2. Everyone involved had a different view of what representative participants would be (demographics, views, experience, etc.) so it was unhelpful. More useful was the concept of inclusiveness, lowering barriers to participation and creating a process accessible to all in a variety of ways. 3. Representativeness is a very difficult concept to define with any level of precision. 4. In qualitative research, representativeness is judged by the participant's experience/knowledge of the phenomenon of interest. This is a completely different issue from representativeness in quant research. 5. 'Representativeness' should be approached with extreme caution as it is rare for people to agree on what participants should be representing: demographics, perspectives, the 'everyman', the experiential expert etc., and thus it can be manipulated. 6. Representativeness is a much more nuanced concept than allowed for in these questions. 7. What are appropriate criteria for representativeness in the context of PPI? In my opinion this could be socio-demographic variables, but perhaps (social, individual) values, (political, religious) orientation or (topic specific) opinions are at least as relevant (and need not coincide with socio-demographic characteristics). | 7 |
| b) | Relevance of representativeness | 1. All depends upon the aim with the activity/research and the research method used 2. Representativeness is important, but it can miss issues of inequality and inequity, the later are in my view extremely important. 3. The importance of representativeness of participants strongly depends on you project/research objectives. 4. Only that selection and the role/relevance of representativeness may be context specific. The issue of proxy/surrogacy is one that arises for pediatric patients/participants. 5. Above you ask how important it is for patients/publics to be representative of the target population - this depends entirely on the specific purpose of the PPI and should not be answered in general 6. As I said in the response about my other paper, this is a big topic, actually several big topics! 7. It is essential to high quality research. There is much guidance on PPI already available, however, there is no harm in receiving more to ensure we do it better 8. I also think we judge the representativeness of patient partners far more harshly than we do other research team mates. For example, a clinician on a project is likely not representative of every other clinician. *   * last quote not counted here as it belongs to quote 8 under c) | 7 |
| c) | Challenges associated with reaching / implementing representativeness | 1. I was not interested in representativeness but rather a range of views in order to better understand the various views that might exist and also to test the online approach to public deliberation. 2. In this case representativeness was not aimed at, which means that also in the conclusions generalization is not possible 3. I'm a bit puzzled by the question about generalisability. We don't necessarily think of PPI as always being concerned with this. It's more about ensuring a range of perspectives are represented in the research. 4. The notion of representativeness in PPI is a load of nonsense and entirely misses the point. Talking about representation deflects from the real value of PPI. No one questions how representative I the researcher am when I conduct a project. Why do we need to question how representative patients are? It's time to move away from this and get on with involving patients in our work. On one hand I feel confident and competent when it comes to PPI. But recently I tried to recruit patients/public for a research initiative (planning a research strategy) and because of the broad nature of the aims of the initiative, I found it hard to think about who to recruit. 5. More you aim for representativeness and more likely you end up doing surveys (with little or no deliberation). Thus, representativeness is not a useful criteria. 6. I think "representativeness" needs to be better defined - I for one would argue that "representative" is not a helpful word to use, as it obscures the focus on experiential knowledge and has connotations with eg. sampling criteria which I think are inappropriate for patient involvement. In the study I've responded about here (hidden carers), we did seek people who self-identified as caring, but I do not think we judged this as about representation or representing. In my experience "representativeness" can be used by researchers to dismiss the views of their public contributors ("they're not representative of my patient group so their opinions don't need to be taken into account"), which is why I have a rather negative view of it! 7. My research is purely qualitative which doesn't aim to be generalizable. 8. Many activities we organized were meant to engage communities who have been traditionally underrepresented, and other activities were meant to engage community leaders. I think any successful PPI has to be thoughtful about who to engage with, which is not always a metric of the overall community population. 9. Difficult to achieve representativeness because people who agree to be interviewed are likely to be different from those who do not. 10. Representativeness can be a difficult concept and thinking about perspectives and purpose of the PPI activity. 11. Seeking 'informed' consent is the biggest barrrier to participation, and hence, representativeness. We found telephone interviews achieve might higher participation rates than postal surveys. Also, having an opt-out system of contacting patients (with an information letter sent prior) achieved a good, and representative result, whereas an opt-in approach would have achieved <20% response rate. 12. I don’t think true representatives is possible. 13. The need for representativeness varied based on my studies, hard to answer in a general term. 14. This study was about patients with complaints. Research shows that complaints that are actually reported are the tip of an iceberg. So we can never be sure about the representativeness of the population. | 14 |
| d) | Challenges and important issues to consider for selecting PPI participants | 1. We conducted our study with an experimental design to maximize internal validity, knowing that there would be limits to external validity. You cannot control generalizability completely because asking laypersons to spend a lot of time on research will inevitably lead to self selection. You just can't get around that, if the subjects are voluntary. 2. It's not good idea to use the same people for PPI to represent a group on different issues. The PPI group should have expertise in the area or topic, and the same people could not be in PPI. 3. The challenges of reaching out and recruiting among vulnerable populations (low socio-economic status, low health literacy, etc.) 4. I am not sure whether the emphasis in this survey was only on representativeness, and not - for instance - on required skills of researchers. Carrying out PPI requires skills that not all researchers have automatically! 5. Selection of participants should not be driven solely by ideas concerning randomness but should use aspects of qualitative judgement also. 6. I think it is important that any patient and public involvement be clearly identified in regards to the research questions and aims. Specifically, why do you want to speak with or engage with this group and how will their contributions help with the research. 7. Make PPI involvement accessible to sign language users. 8. It is essential that the *differences* between patients and publics be understood. As well, we need a third category - "community" which identifies a different public constituency with collective interests that also warrant engagement and representation. 9. It requires a lot of people, so you have to plan for low recruitment; we used an experimental model to ensure we obtained at least internally valid results. 10. Racial and ethnic representation is an often-overlooked area. 11. Include all age groups, all SES levels. 12. I think there is both geographical community and a disease-specific community that needs to be consulted. 13. There are many resources (e.g., INVOLVE, PCORI, many in the academic literature) that are available to researchers and others who want to learn about how best to recruit for PPI work. It simply takes some hard thinking about your purpose and some sleuthing through the literature for examples of techniques; I also think we judge the representativeness of patient partners far more harshly than we do other research team mates. For example, a clinician on a project is likely not representative of every other clinician. | 13 |
| e) | Role and relevance of PPI | 1. This article was conducted 8-9 years ago before the great focus on PPI - today I would focus more on PPI and construct the research differently trying to involve dropouts more though the study process. 2. To me PPI is the participation in the design and execution of a project, to differentiate this from participating as a subject. 3. Each person has a unique experience so very hard to capture this is PPI. However, I believe that the concept of researchers and practitioners being open to this is very important even though it may not give an insight into everyone's view | 3 |
